# Lipid Membrane-Coated Nanopipettes for Enhanced Resistive Pulse Sensing of Exosomes

**DOI:** 10.1021/acsami.6c02193

**Published:** 2026-03-23

**Authors:** Kaoru Hiramoto, Chisae Kaji, Ayumi Hirano-Iwata

**Affiliations:** † Frontier Research Institute for Interdisciplinary Sciences, 13101 Tohoku University, 980-8578 Aramaki-aza-Aoba, Sendai, Japan; ‡ Research Institute of Electrical Communications, Tohoku University, 980-8577 Katahira, Sendai, Japan; § School of Engineering, Tohoku University, Aramaki-aza-Aoba 980-8579, Japan; ∥ Advanced Institute for Materials Research, Tohoku University, 980-8577 Katahira, Sendai, Japan

**Keywords:** exosomes, nanopore sensing, resistive pulse sensing, lipid membrane coating, electrokinetic transport, interfacial functionalization

## Abstract

We report the use of lipid membrane-coated quartz nanopipettes to enhance exosome detection via resistive pulse sensing. By exploiting the self-assembly and compositional versatility of lipid molecules, nanopipettes were functionalized with lipid membranes comprising neutral and cationic lipids, with or without cholesterol, to modulate surface charge and membrane viscosity. Using bovine milk–derived exosomes as a model system, we demonstrate a marked improvement in capture rate and a reduction in nonspecific adsorption. This improvement further enabled statistical analysis of translocation times and signal amplitudes, providing significant insights into the interactions between exosomes and lipid membranes during nanopore passage under an applied electric field.

## Graphical abstract

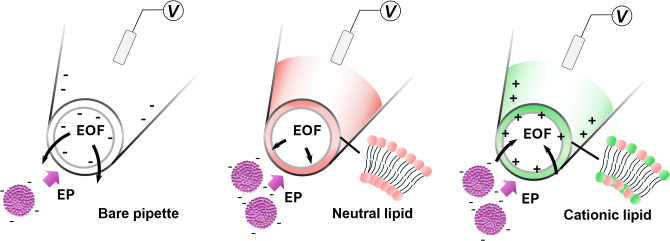

## Introduction

Extracellular vesicles (EVs) are membrane-bound particles secreted by nearly all cell types as a means of intercellular communication.^1^ Among them are exosomes, typically classified as small EVs ranging from 30 to 200 nm, which are generated through the endocytic pathway.^2^ Due to the diverse molecular cargos they carry and their role in mediating cell-to-cell communication, exosomes have attracted significant attention not only in basic cell biology but also as promising biomarkers^3^ and therapeutic targets in clinical diagnostics.^4^

To characterize EVs and elucidate their biological functions, numerous analytical techniques have been developed. Ensemble-averaged methods such as dynamic light scattering (DLS)^5^ and nanoparticle tracking analysis (NTA),^6^ provide size distribution and concentration measurements.^7^ In contrast, transmission electron microscopy (TEM), atomic force microscopy (AFM), and super-resolution microscopy allow the observation of individual EVs, providing insights into their heterogeneity and morphological features.^8^ While these technologies have greatly facilitated EV analysis, the ongoing development of innovative methods is vital to advancing EV research. One promising technique in this regard is nanopore sensing,^9^ which enables label-free detection of nanoscale particles by measuring ionic current modulations as individual particles translocate through a nanopore.^10^ Due to its relatively simple setup and detection principle, pore functionalization and the control of the flow environment in the vicinity of the pore is critical to enhance capture rate and resolve the behavior of single-nanoparticle translocations.^11^ With regard to EV sensing, tunable resistive pulse sensing (TRPS) adjusts pore size by mechanically stretching the nanopore membrane,^12^ and the use of low-aspect-ratio nanopores^13^ as well as nanofluidic chips for filtration and differentiation of EVs^14^ can improve signal resolution. In addition, the application of salt gradients^15^ has been shown to be an effective way to increase capture rate, while the incorporation of ligands, such as aptamers, into nanopores can improve specificity.^16^

From a biological perspective, exosome transport is inherently linked with membrane–membrane interactions, given their origin from cellular membranes and uptake by recipient cells.^17^ Inspired by this, we explored the use of lipid membrane coatings on glass nanopipettes to mimic biological interfaces and potentially enhance exosome translocation signals through solid-state nanopores.^18^ Pioneering work was done by Mayer’s group, which demonstrated the detection of amyloid-β species using antibody-functionalized lipid membranes on silicon nitride nanopores.^19^ Similarly, Keyser’s group reported enhanced λ-DNA translocation using lipid-coated nanocapillaries.^20^ More recently, Chen et al. investigated protein-vesicle affinities within confined lipid environments formed inside a glass capillary.^21^ Despite these advances, the application of lipid coatings for detecting larger biomolecular assemblies such as exosomes remains largely unexplored.^22^

In this study, we utilized quartz nanopipettes and functionalized them with lipid membranes (Figure 1a). Specifically, we used 1,2-dioleoyl-*sn*-glycero-3-phosphocholine (DOPC), a neutral glycerophospholipid commonly used to mimic biological membranes, and 1,2-dioleoyl-3-trimethylammonium-propane (DOTAP), a synthetic cationic lipid, to tune the nanopipette surface charge. Furthermore, cholesterol was added to DOPC-DOTAP mixtures to examine its effect on membrane fluidity, as it is known to decrease membrane fluidity when combined with unsaturated phospholipids^23^ (Figure 1b). The lipid membrane was coated on the tip of the nanopipette via spontaneous rupture and fusion of liposomes containing the above lipids (vesicle fusion), and this method allowed facile change in the surface charge of pipette walls, as well as giving a low-adhesive surface to prevent nonspecific adsorption (Figure 1c). We used bovine milk–derived exosomes as a model target. These exosomes contain microRNAs and mRNAs,^24^ as well as typical exosomal markers such as CD81, CD63, and CD9.^25^ Owing to their high biocompatibility and protein-rich composition,^26^ milk-derived exosomes are considered a promising natural resource for therapeutic applications and drug delivery systems.^27^ We demonstrated the significant improvement in exosome translocations on lipid membrane-coated nanopipettes, which further enabled the investigation of exosome–membrane interactions that produced differences in translocation dwell times across various lipid mixtures. This study presents a facile approach to improve exosome detection in RPS systems, highlighting the potential of artificial lipid membranes for nanopore-based biosensing.

**Figure 1:**
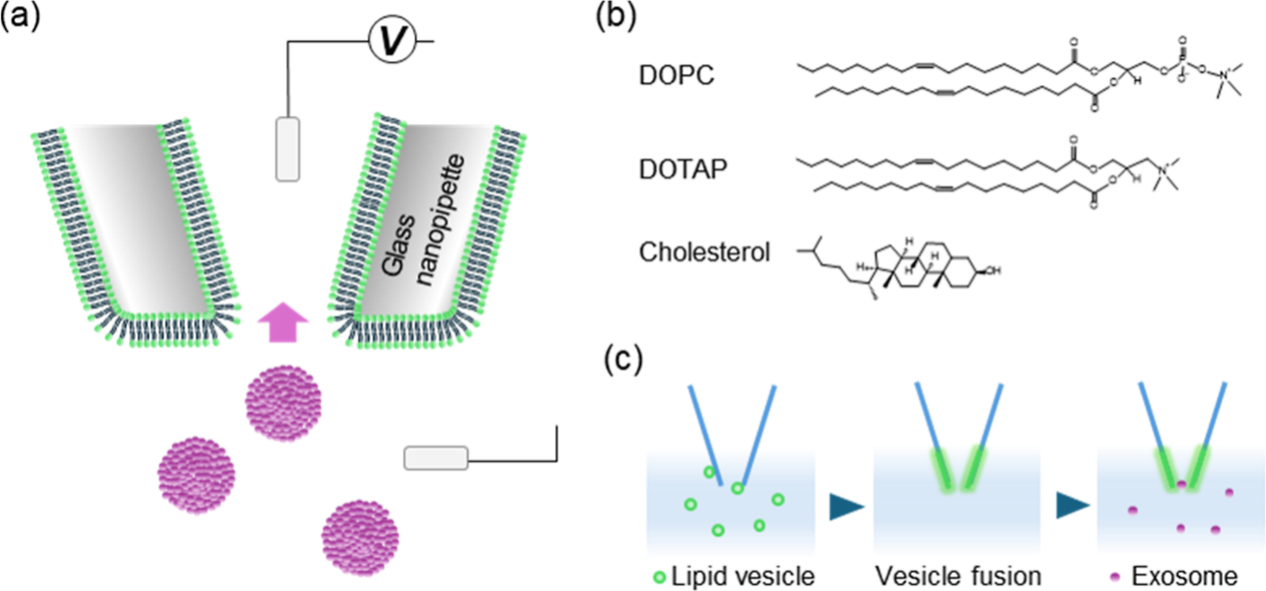
Lipid-membrane-coated nanopipette for exosome detection. (a) Schematic illustration of the detection principle. (b) The lipid molecules used in this study. (c) Experimental workflow for nanopipette coating and exosome detection.

## Results and Discussion

### Fabrication and Characterization of Lipid Membrane-Coated Nanopipettes

Quartz nanopipettes were fabricated using a laser-assisted pulling. To enable the translocation of exosomes up to 200 nm in diameter, pulling parameters were adjusted to produce nanopores larger than this threshold. Scanning electron microscopy revealed an average tip opening of 289 ± 32 nm (*n* = 5; Figure 2a), while conductance-based estimates indicated a mean pore size of 226 ± 33 nm (*n* = 16; Table S1, see eqs S1 for the derivation).

**Figure 2:**
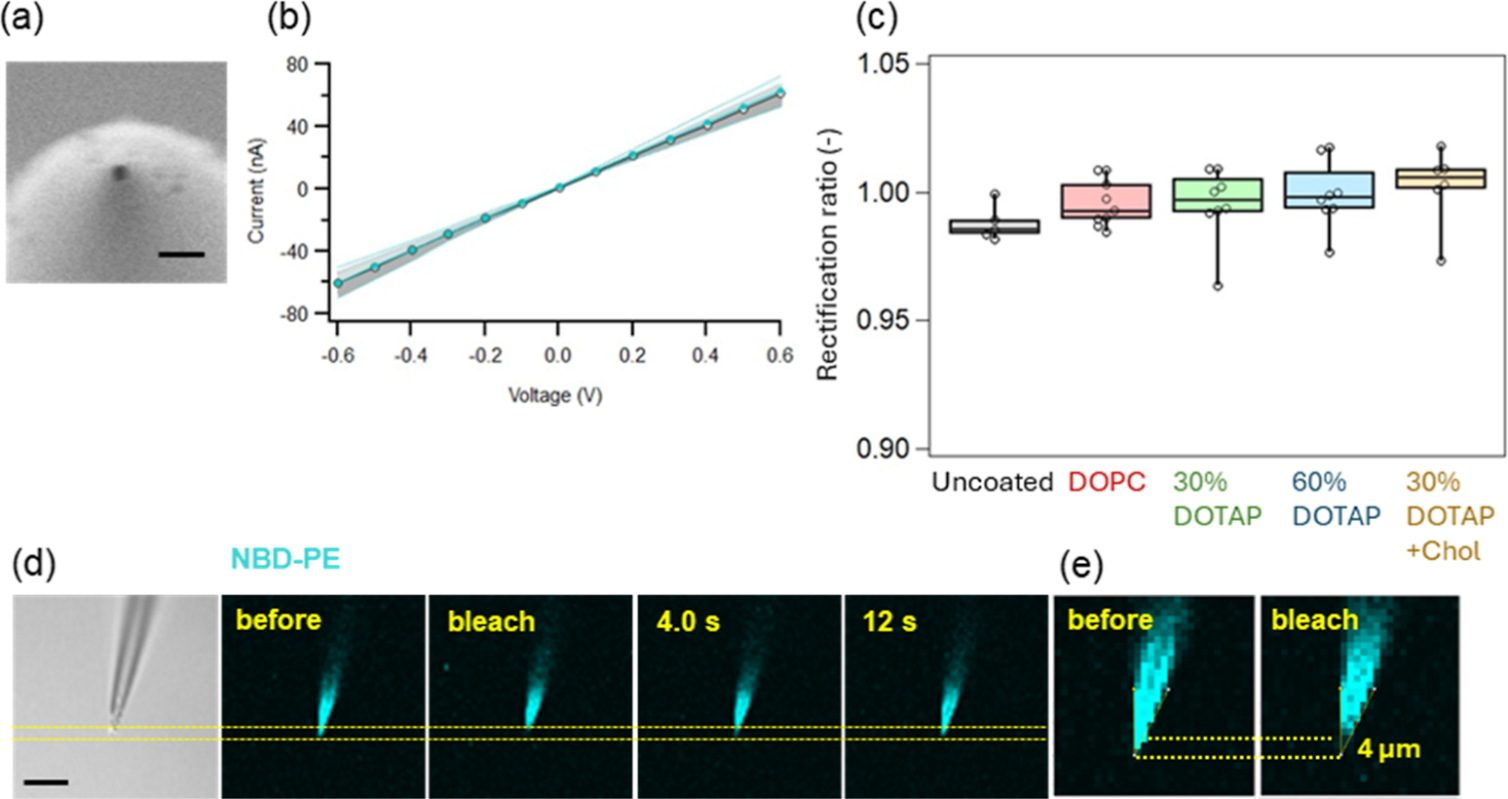
(a) SEM image of a representative nanopipette (scale bar: 1 μm). (b) Current–voltage (*I*–*V*) curves obtained before (black) and after (cyan) coating the tip with a DOPC lipid membrane. Circles represent mean currents from *n* = 3 pipettes, and shaded regions denote the standard deviations. (c) Rectification ratios of uncoated and lipid membrane–coated nanopipettes. *n* = 5 for uncoated, *n* = 9 for DOPC-coated, *n* = 8 for 30% and 60% DOTAP-coated, and *n* = 6 for 30% DOTAP + Chol–coated nanopipettes. (d) Bright-field and fluorescence images of a DOPC membrane–coated nanopipette during FRAP measurements (Ex: 458 nm, Em: 476 nm). The argon laser power was set to 100%, and the pipette tip was photobleached for 300 ms, followed by sequential image acquisition at 2 s intervals. Scale bar: 10 μm. (e) Magnified images of the nanopipette tip before and after photobleaching. A circular region with a diameter of 4 μm at the tip was photobleached.

Lipid membranes were coated onto the pipette wall via spontaneous vesicle fusion. To investigate the effect of lipid membrane compositions on the modulation of the pipette surface, four variations of liposomes were prepared by extrusion: vesicles consisting of a neutral lipid (DOPC) and DOPC mixed with a cationic phospholipid (DOTAP) at concentrations of 30 and 60 mol % (hereafter referred to as 30% DOTAP and 60% DOTAP, respectively). In addition, a DOPC–DOTAP–cholesterol mixture with a molar ratio of 40:30:30 was also prepared (hereafter referred to as 30% DOTAP + Chol). To all lipid mixtures, 1 mol % of 1,2-dioleoyl-*sn*-glycero-3-phosphoethanolamine-*N*-(7-nitro-2–1,3-benzoxadiazol-4-yl) (NBD-PE) was added so that the lipid coatings could be imaged using fluorescence microscopy. The liposomes were characterized by DLS, which showed an average diameter of 92 nm and zeta potentials of 2.1 mV for DOPC liposomes and over 20 mV for DOTAP-containing liposomes (Table S2). Figure 2b show the representative current–voltage (*I*–*V*) curves obtained before (black) and after (cyan) coating the nanopipette tip with a DOPC lipid membrane. Since the surface charge of quartz capillaries is negative, the uncoated pipette exhibited negative rectification, whereas after DOPC coating, this effect was neutralized. Figure 2c summarizes the rectification ratios for nanopipettes with different lipid membrane compositions. The uncoated pipettes showed a mean rectification value of 0.990, while the rectification ratios slightly increased with lipid membrane coating in the order of DOPC (0.995), 30% DOTAP (1.001), 60% DOTAP (1.002), and 30% DOTAP + Chol (1.006).

We also performed fluorescence recovery after photobleaching (FRAP) analysis^28^ on the tip of the nanopipettes. Figure 2d,e show several selected images of the tip coated with a DOPC membrane during the FRAP measurement, which exhibits quick fluorescence recovery after the pipette tip was photobleached. The diffusion coefficient of each lipid mixture is summarized in Figure S1 and Table S3 (see eqs S2–S4 for the derivation). The DOPC membrane showed the highest fluidity, whereas the introduction of 30% DOTAP decreased it, likely due to stronger electrostatic interactions between the cationic lipid head groups and the negatively charged pipette surface. The addition of cholesterol to 30% DOTAP slightly increased the membrane fluidity, suggesting a reduction of this effect. Although the diffusion coefficient of the lipid membranes on the pipette tip was smaller than that reported on a glass surface (1–4 μm^2^/s^29^), the observed fluorescence recovery supports the formation of a continuous lipid membrane rather than mere adhesion or aggregation of lipid molecules on the pipette wall.

### Lipid Membrane Coatings Increased the Capture Rate of Exosomes

The exosome was detected after thorough washing of excess lipid vesicles with DPBS. By following the washing procedure, residual liposomes or dissociated lipid molecules did not generate detectable blockade signals (Figure S2). Figure 3a shows representative current traces from uncoated and DOPC membrane-coated nanopipettes at multiple applied voltages. The corresponding peak current (Δ*I*) and dwell-time histograms are summarized in Supporting Information (Figure S3). For the DOPC-coated nanopipette, the peak current increased with increasing |*V*| under both positive and negative biases, while the dwell time decreased moderately at higher voltages. The capture rates are summarized in Figure 3b, showing that exosomes were rarely detected with uncoated nanopipettes, whereas capture rates increased significantly with lipid membrane coatings, particularly under positive voltage. Since *I*–*V* characteristics of coated pipettes showed rectification close to 1 (Figure 2c), this polarity dependence of capture rate is attributed primarily to electrokinetic transport asymmetry at the pore rather than ionic conductance rectification within the nanopipette. To account for pore-to-pore variability, capture rates were additionally normalized by nanopipette conductance (*G*), obtained from individual *I*–*V* measurements. The normalized results (Figure S4) exhibited the same coating-dependent trend, confirming that the enhancement is independent of pore geometry.

**Figure 3:**
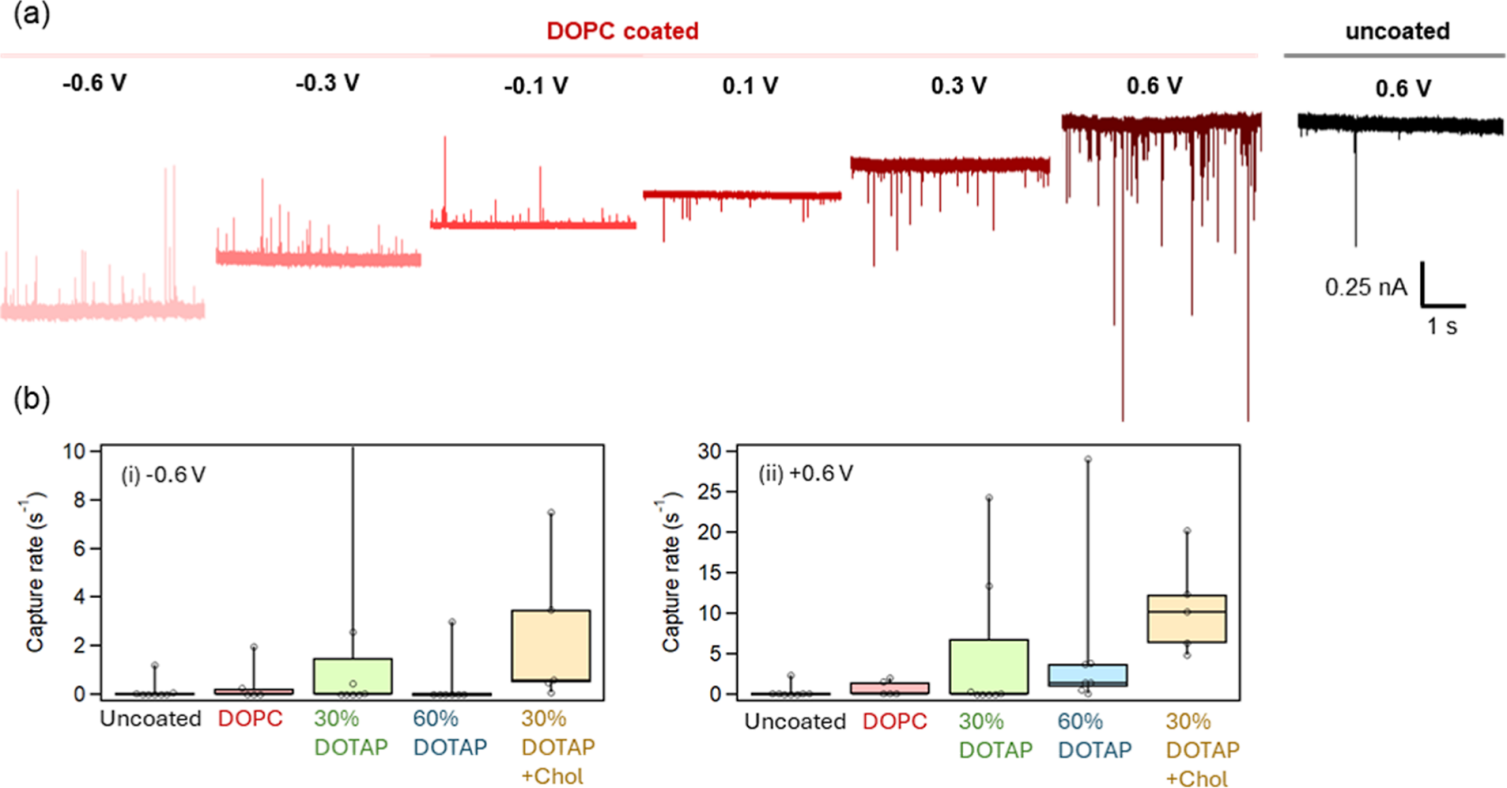
Translocation events of milk exosomes (a) Representative current traces of exosome translocation with DOPC membrane-coated pipette at multiple applied voltages and uncoated pipette at 0.6 V. (b) Capture rates of exosomes with uncoated and the lipid membrane-coated nanopipettes. The capture rate was determined by normalizing the number of signal events recorded over 60 s from each measurement. Applied voltage: (i) *V*_in_ = −0.6 V, (ii) *V*_in_ = 0.6 V.

### Electrokinetic Driving Forces for Exosome Entry into Lipid-Membrane-Coated Nanopipettes

For the translocation of exosomes through the nanopore, electrophoresis (EP) and electroosmotic flow (EOF) can act as the primary driving forces.^30^ The effective velocity for inward transport of exosomes can be expressed as eqs 1 and 2

1 $$v_{eff}=\left(\mu_{EP}+\mu_{EOF}\right)E$$

According to the Smoluchowski relation, the electrophoretic and electroosmotic mobilities^31^ are given by

2 $$\mu_{EP}=\frac{\varepsilon \zeta_{p}}{\eta },,\mu_{EOF}=-\frac{\varepsilon \zeta_{w}}{\eta }$$

where ε and η are the permittivity and viscosity of the solution, respectively, ζ_p_ is the zeta potential of the particle, ζ_w_ is the zeta potential of the nanopore wall, and *E* is the applied electric field. The zeta potential of the milk exosomes was measured to be −10 ± 3.4 mV, indicating that the exosomes migrate toward the pipette via EP when a positive voltage is applied inside the pipette.

Taking the DOPC-coated pipette as an example, DOPC is a zwitterionic lipid and is therefore expected to substantially reduce the effective negative surface charge of the glass nanopipette, thereby diminishing EOF contributions. Under these conditions, electrophoretic transport of negatively charged exosomes is expected to dominate the net motion.

To further verify whether the observed exosome translocation is governed by electrokinetic transport, recordings were performed under different ionic strengths using DOPC-coated nanopipettes (low salt: 1× DPBS; high salt: 10× DPBS; Supporting Information, Figure S5). Under high-salt conditions, longer dwell times with broader distributions were observed, whereas under low-salt conditions the dwell times were shorter and the distributions narrower, accompanied by higher capture efficiency. This behavior is consistent with electrokinetic transport mechanisms. At lower ionic strength, reduced charge screening increases the Debye length and enhances electrophoretic driving forces, facilitating more efficient capture and faster translocation of exosomes. In contrast, at higher ionic strength, electrostatic interactions are screened, suppressing electrophoretic mobility and increasing the relative contribution of diffusion and pore–vesicle interactions, which leads to longer and more broadly distributed dwell times.

The capture rate at *V*_in_ = 0.6 V increased in the order of DOPC, 30% DOTAP, 60% DOTAP, and 30% DOTAP + Chol, partially consistent with the zeta potentials measured for liposomes with these compositions (Table S2). Increasing the cationic charge at the nanopore interface through lipid membrane coating can modify both the magnitude and direction of electroosmotic flow (EOF), thereby enhancing the net inward electrokinetic transport and improving capture rate. Similar electrokinetic transport behavior has been reported in functionalized nanopores, where surface charge and interfacial properties regulate nanoparticle motion under applied electric fields.^11^ Although the rectification change observed in lipid membrane–coated nanopipettes was small, these results indicate that even slight modulation of surface charge can significantly influence capture rate.

### Antifouling and Stabilizing Effects of Lipid Membranes

We investigated changes in current noise before and after lipid membrane coating, as well as after exosome detection, to assess the measurement performance of lipid membrane-coated nanopipettes.^32^ We evaluated baseline current noise (RMS) over repeated measurements (∼20 min). In uncoated nanopipettes, exosome detection produced a pronounced increase in baseline fluctuations, whereas lipid membrane–coated nanopipettes maintained stable currents over the measurement period (Figure S6). To further investigate the origin of the noise, power spectral density (PSD) analysis was performed (Figure 4a–e). PSD analysis provides quantitative information on the frequency-dependent noise characteristics of the nanopore system.^33^ In general, low-frequency noise is associated with surface conductance fluctuations and interfacial instability,^34^ whereas high-frequency noise primarily arises from thermal and dielectric noise originating from both the nanopore and the recording system. Within the recording bandwidth of 10–100 kHz, the PSD spectra showed negligible changes across all conditions, suggesting that neither lipid membrane coating nor exosome detection significantly affected the recording noise. In contrast, in the 1–10 kHz range, the PSD slope of uncoated nanopipettes increased markedly after exosome detection, suggesting enhanced surface conductance fluctuations. This behavior is likely due to nonspecific adsorption of exosomes onto the glass surface, which alters the local electrical environment of the pore. For lipid membrane–coated nanopipettes, although low-frequency noise slightly increased after coating—consistent with the formation of a lipid layer—the additional increase following exosome detection (denoted as “After” in Figure 4) was substantially smaller than that observed for uncoated pipettes. This result suggests that lipid membrane coating effectively suppresses exosome adsorption onto the pipette wall. The changes in the slope of the PSD (1–10 kHz) are summarized in Figure 4f. The reduction in slope after exosome detection was most pronounced for the 30% DOTAP + Chol membrane. Notably, the slope in the very low-frequency region (<10 Hz), commonly referred to as flicker noise,^34^ decreased particularly in the presence of cholesterol, indicating that cholesterol enhances interfacial stability and mitigates electrical fluctuations at the nanopipette tip. Importantly, the reduced low-frequency noise correlated with improved exosome capture rate (Figure 3b), suggesting that lipid membrane coating enhances sensing performance by stabilizing the electrical interface and minimizing signal loss. Although clogging events were still observed with lipid membrane–coated nanopipettes, these events occurred randomly and were not systematically correlated with the coating conditions. This suggests that lipid membrane coatings reduce the probability of adsorption-induced blockage but do not completely eliminate it (Figure S7).

**Figure 4:**
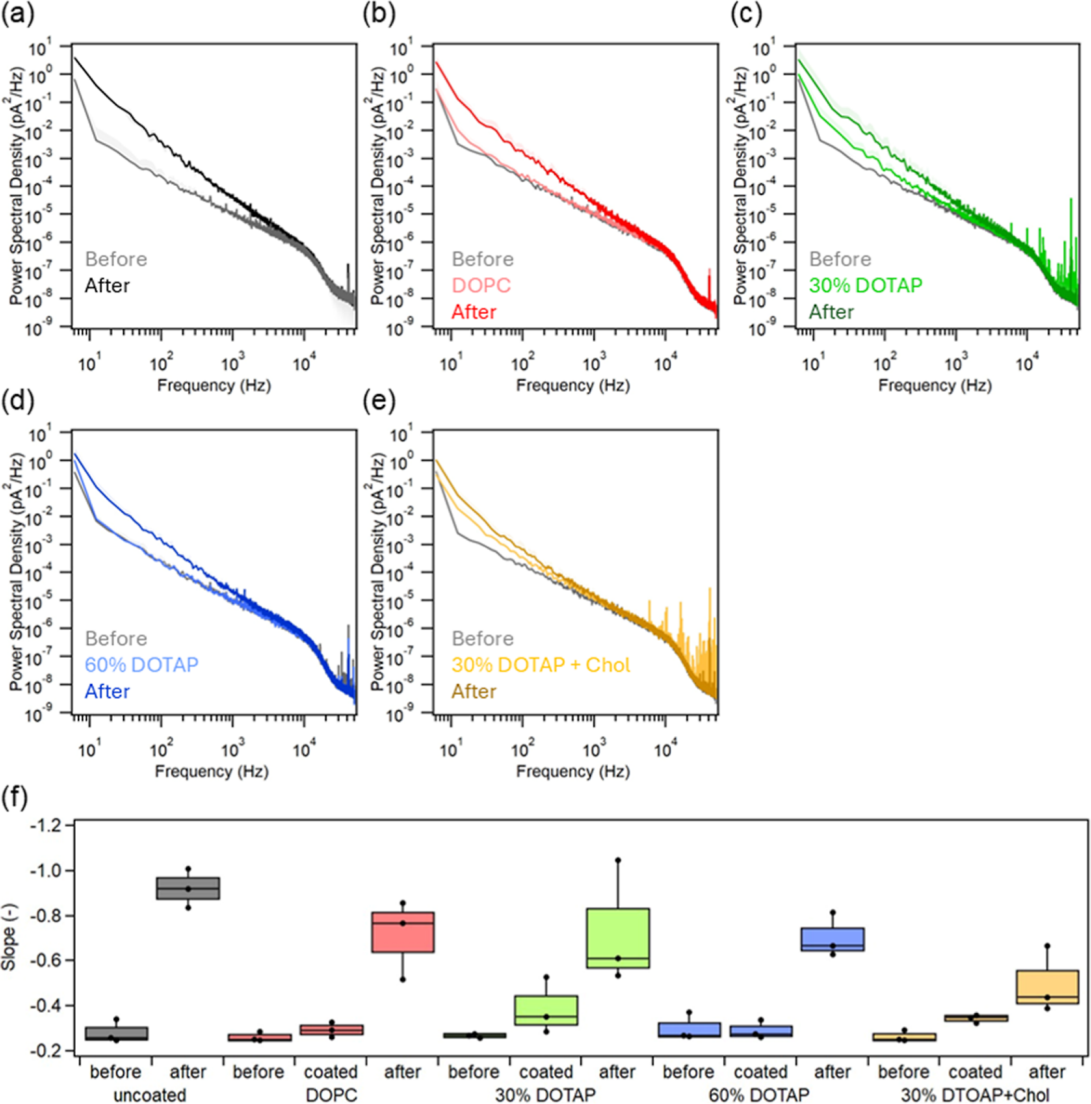
Noise analysis (a) Power spectral densities before and after the detection of exosomes with uncoated pipettes. (b–e) Power spectral densities before and after the coating of DOPC, 30% DOTAP, 60% DOTAP, and 30% DOTAP + Chol membranes, respectively, as well as after the detection of exosomes with each pipette. (f) The trends of PSD slopes before and after the lipid membrane coatings, as well as after the detection of exosomes. The curves of PSD in the range of 1–10 kHz were fitted using the least-squares method.

We also attempted to observe exosomes using fluorescence microscopy. The exosomes were stained with a deep-red fluorescence membrane marker (Exo Sparkler) and introduced into the well using either uncoated or 30% DOTAP-coated pipettes. Figure 5a shows the selected images of the FRAP measurement on the tip of the pipettes after 30 min of exosome introduction. We observed deep-red fluorescence in/on the pipettes. Interestingly, the point excitation of the Exo Sparkler dye completely bleached the exosomes on the uncoated pipettes, while a certain level of fluorescence recovery was observed on the lipid membrane-coated one (Figure 5b). These results suggest that exosomes on uncoated pipettes may be strongly adhered to the pipette wall, whereas exosomes on the lipid membrane coated pipettes had some mobility to translocate into the pipette. This result also supports the antifouling effect of the lipid membrane coating.

**Figure 5:**
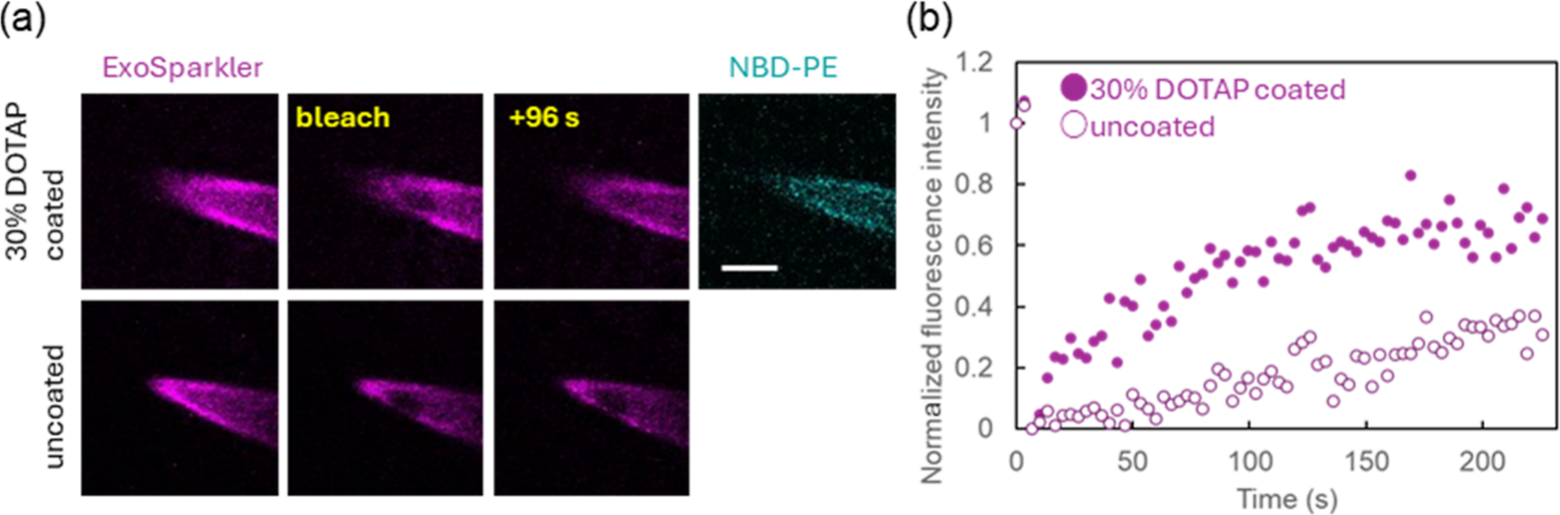
FRAP analysis at the tips of nanopipettes. (a) FRAP images of nanopipettes either uncoated or coated with 30% DOTAP. Deep-red fluorescence (excitation: 633 nm, emission: 668 nm) indicates the presence of exosomes in or on the nanopipettes, while cyan fluorescence (excitation: 458 nm, emission: 476 nm) corresponds to NBD-PE in the lipid membrane. Photobleaching was performed for 400 ms using a 633 nm laser with 100% power. Scale bar: 10 μm. (b) Time-course plot of normalized fluorescence intensity on uncoated and 30% DOTAP-coated nanopipettes.

#### Signal Characteristics of Exosome Translocation through Lipid-Coated Nanopipettes

Representative translocation signals are shown in Figures 6a and S8, which exhibited a typical triangular shape characteristic of spherical particles. Figure 6b,c summarize the Δ*I*/*I* and dwell-time histograms at +0.6 V, and Figure 6d illustrates the proposed mechanism, allowing systematic comparison of exosome translocation among different lipid-membrane compositions. The histograms recorded at −0.6 V are presented in the Supporting Information (Figure S9), and the composition-dependent trends are consistent with those observed at +0.6 V. The most probable values of Δ*I*/*I* and dwell time were obtained by fitting the corresponding histograms with either Gaussian or log–normal distributions and are summarized in Table 1 (see eqs S5–S8 for the derivation). Statistical analysis could not be performed for events recorded with uncoated pipettes at −0.6 V due to the low capture rate and broad distribution of the signals.

(1) Comparison between neutral and cationic lipids

**Figure 6:**
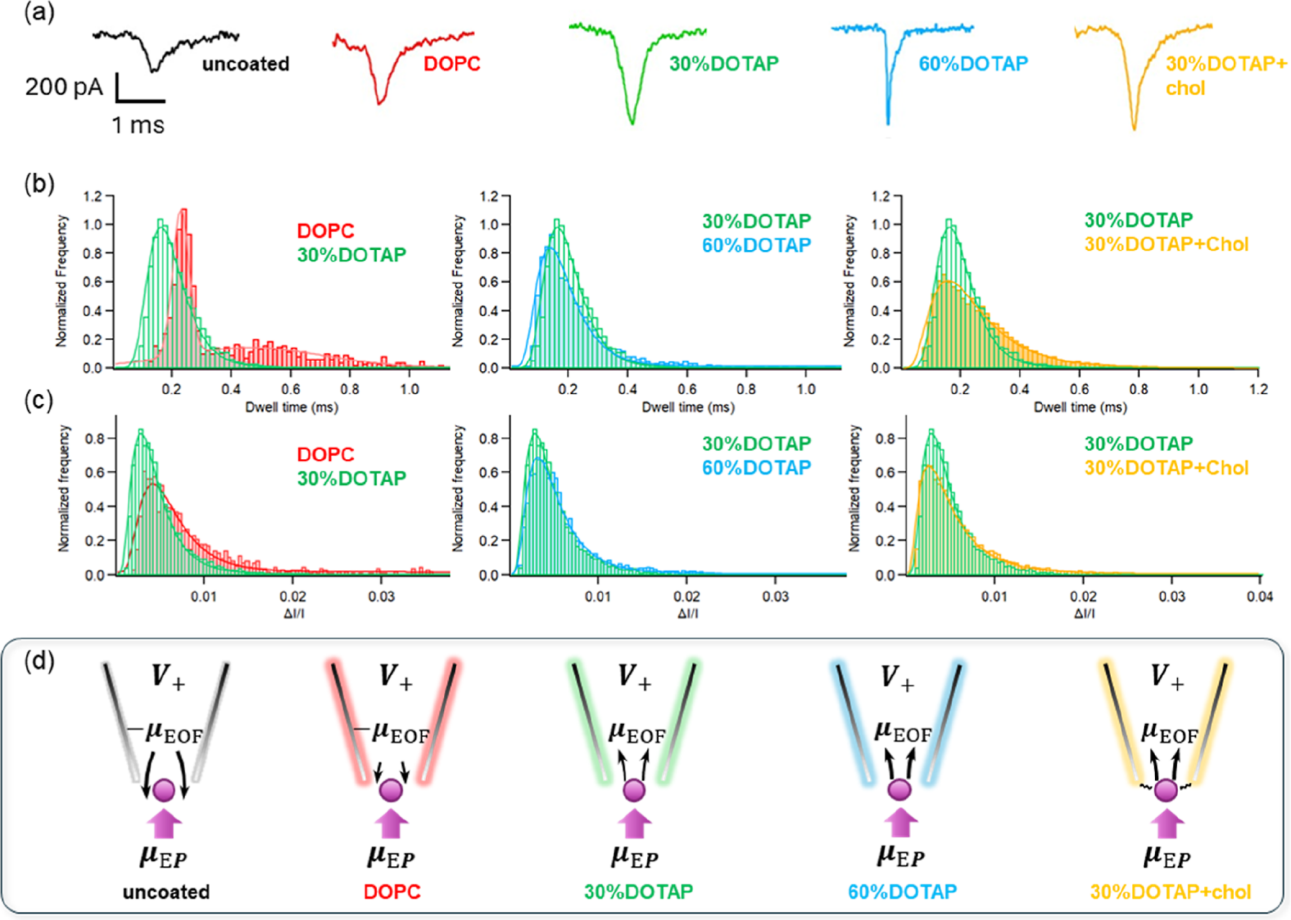
Exosome translocations with uncoated and lipid-membrane coated pipettes. (a) Typical events. (b) Histograms showing the trend of normalized peak height (Δ*I*/*I*) (bin-width: 0.004) and (c) dwell time (bin-width: 16 μs) of translocation signals under *V* = +0.6 V. (d) Schematic illustrations of possible electrokinetic forces occurring at the pipette tips.

**Table 1:** Most Probable Values of Normalized Peak Height and Dwell Time under +0.6 and −0.6 V on the Uncoated and Lipid Membrane-Coated Pipettes

	+0.6 V		–0.6 V	
	*ΔI*/*I*	dwell time (μs)	*ΔI*/*I*	dwell time (μs)
uncoated	0.022 ± 0.005	336 ± 19	-	-
DOPC	0.042 ± 0.008	230 ± 0.6/465 ± 17	0.033 ± 0.004	170 ± 2.4
30% DOTAP	0.028 ± 0.002	163 ± 0.7	0.023 ± 0.002	214 ± 1.2
60% DOTAP	0.031 ± 0.003	138 ± 1.3	0.022 ± 0.005	322 ± 4.4
30% DOTAP + Chol	0.025 ± 0.004	159 ± 1.6	0.014 ± 0.001	364 ± 2.7

Based on the electrokinetic transport model in eq 1, neutral DOPC is expected to reduce the effective wall charge compared with bare glass, thereby diminishing outward EOF. However, the relatively broad distribution in the dwell-time histogram suggests that this surface modification remained relatively weak and heterogeneous. The dwell-time histogram for DOPC was well fitted by a two-Gaussian distribution. We attribute the first population (mean dwell time = 230 μs) to exosome entry primarily driven by electrophoresis (EP), while the second population (mean dwell time = 465 μs) likely reflects partial interference from residual EOF or heterogeneous interfacial conditions. In contrast, the 30% DOTAP membrane exhibited faster translocation events, suggesting that the surface charge modification reversed the opposing EOF. The more unified Δ*I*/*I* distribution observed for 30% DOTAP also indicates a more stable electrostatic environment at the nanopipette interface.

(2) Increasing the DOTAP concentration

We initially assumed that increasing the DOTAP concentration would lead to faster and more pronounced translocation signals due to stronger electrostatic attraction. However, only modest changes in dwell-time and Δ*I*/*I* distributions were observed between 30% and 60% DOTAP. One possible explanation is that the distribution of DOTAP between the inner and outer leaflets of the supported lipid membrane is not well-defined. Charged lipids may redistribute on the nanopipette surface to form an asymmetric bilayer structure, which could limit the surface charge modulation achieved solely by increasing DOTAP concentration.

(3) Incorporation of cholesterol

Incorporation of cholesterol into the 30% DOTAP membrane resulted in longer translocation times. Although FRAP measurements showed a slightly higher lipid diffusion coefficient for 30% DOTAP + Chol than for 30% DOTAP, FRAP mainly probes lateral lipid diffusion and does not directly reflect the interfacial viscosity experienced by translocating vesicles. In the supported membrane geometry used here, cholesterol likely stabilizes lipid packing and modulates electrostatic and steric interactions at the membrane–vesicle interface. Therefore, the prolonged dwell times observed for the DOTAP + Chol membrane are more likely associated with strengthened and more uniform interfacial interactions between the lipid membrane coating and the exosome surface.

Consequently, the lipid membrane–coated pipette not only provided improved capture rates and reduced signal loss (antifouling effect) for exosomes, but also enabled the extraction of unique information on the forces involved (EP and EOF) and the interactions between exosomes and lipid membranes during translocation.

## Conclusion

In this study, we proposed lipid membrane coatings to enhance the efficiency of exosome detection via resistive pulse sensing with glass nanopipettes. By incorporating the cationic lipid DOTAP, the surface charge of the pipette wall was rendered positive, thereby increasing the electrostatic attraction toward negatively charged exosomes. The addition of cholesterol provided antifouling properties with prolonged dwell time. In the present study, purified milk-derived exosomes were spiked into DPBS, representing a relatively ideal detection condition. Future studies should evaluate exosomes derived from biological fluids and clinically relevant samples to further assess the applicability of lipid membrane–coated nanopipettes in more complex environments. Compared to other exosome detection techniques, lipid membrane coating offers advantages in preparation simplicity, owing to the self-assembling nature of lipid molecules. By rational selection of lipid constituents—such as chain length, headgroup charge, degree of unsaturation, and molecular recognition motifs—the physicochemical properties of the nanopore interface can be systematically tuned to accommodate diverse fluidic environments. Beyond tuning electrostatic interactions, lipid membranes provide a modular platform capable of integrating functional components, including ligands, receptors, and membrane proteins.^35^ Such compositional flexibility enables interfacial engineering for selective vesicle capture and single-particle sensing. Moreover, this biomimetic interface offers opportunities to investigate lipid–biomolecule interactions at confined nanoscale interfaces, potentially advancing the development of responsive and highly selective sensing platforms.

## Materials and Methods

### Reagents

All phospholipids used in this study were purchased from Avanti Polar Lipids, Inc. (United States). Cholesterol was purchased from Wako Pure Chemical, Japan and used after purification. Milk exosome from bovine was purchased from Cosmo. Bio Co., Ltd., Japan. A 10× Dulbecco’s phosphate-buffered saline (DPBS; Nacalai Tesque, Japan) was diluted 10-fold using distilled water and filtered through cellulose acetate membrane (ADVANTEC© pore size 0.20 μm, Toyo Roshi Kaisha, Ltd., Japan) prior to use.

### Preparation of Liposome Solutions

A specified amount of lipids was transferred to a glass vial (Wako Pure Chemical) to achieve a total lipid concentration of 4 mg/mL in chloroform. The chloroform was evaporated by gently circulating nitrogen gas over the solution while rotating the vial, depositing a lipid film on the inner wall. The lipid film was then placed overnight in a vacuum desiccator to remove residual chloroform. The following day, the film was rehydrated with 2 mL of DPBS for 1 h, resulting in a 2 mg/mL lipid solution. The solution was voltexed for 1 min and stored at −80 °C until further purification.

Liposomes of specific lipid compositions were prepared through freeze–thaw cycles and subsequent extrusion. The lipid vesicle solution was heated to room temperature and vortexed for 1 min. The vial was then immersed in liquid nitrogen for 3 min, followed by immersion in warm water (40–60 °C) for 4 min. This cycle was repeated five times. The solution was then extruded using a mini-extruder (Avanti Polar Lipids) with a 100 nm pore size filter (Avanti Polar Lipids), following the manufacturer’s instructions. This extrusion process was repeated 11 times to obtain a liposome solution. The prepared liposome solution was stored at 4 °C and utilized within 2 weeks postextrusion.

### Nanopipette Preparation

Quartz capillaries (O.D. One mm, I.D. 0.5 mm, QF100-50-7.5; Sutter Instrument, United States) were plasma treated for 30 min before the pulling. A CO_2_ laser puller (P-2000, Sutter Instruments) was used to drawn down the capillaries. Parameters of the setting are shown in Table 2. Note that the optimum parameters will vary depending on the machine and environment.

**Table 2:** Laser Puller Settings

	heat	filament	velocity	delay	pull
1st	575	3	35	145	75
2nd	425	0	15	128	200

### Lipid Membrane Coating on a Pipette Tip

The capillary was backfilled with DPBS and the tip was immersed in a DPBS bath of 100 μL. Ag wires (diameter: 0.3 mm) were chlorinated (Ag/AgCl) and inserted into the other opening of the capillary and the bath. First, ionic current versus applied voltage (IV) curves were obtained by recording the current in 0.1 V increments from −0.6 to 0.6 V for 0.5 s each to check the initial state of the capillary. Then 200 μL of the liposome solution prepared to a concentration of two-tenth of the original was added (giving a total concentration of 0.1 mg/mL of liposomes in the bath) to allow the vesicles rupture and fuse on the tip of the capillary to form a bilayer. After 20 min, the excess liposomes were washed by repeating the exchange of the half of the solution with 4 mL of DPBS, in order not to dry out the tip of the capillary. IV curves and constant voltage measurements at −0.6 and 0.6 V were performed between each process to check the rectification and clogging.

### Ionic Current and Resistive Pulse Recordings

The exosome solution was added to the bath to a concentration of four-tenth of the original solution (2.4 × 10^12^ particles/mL). The ionic current was measured by a patch-clamp amplifier (HEKA elektronik, Germany) and recorded using a software Patch Master (sampling rate 100 kHz with a low-pass filter at 10 kHz). The current data was analyzed using Igor Pro 9 software (WaveMetrics, United States). To construct a baseline, a low-pass filter (cutoff frequency: 1 kHz) was applied to the recorded current traces to remove high-frequency components. After filtering, a moving median with a window size of 500 data points was applied to the processed signal to define the baseline. The signals larger than 30% of the baseline current were extracted as the translocation events. This threshold was selected to balance sensitivity and false-positive suppression, and robustness to reasonable threshold variation is demonstrated in the Supporting Information (Figure S10). The peak amplitude was calculated as the subtraction of the maximum current of an event and the baseline, and the dwell time was measured as the full width half-maximum of the current reduction.

### Fluorescence Imaging of Pipette Tips and Exosomes

Fluorescence imaging and fluorescence recovery after photo bleaching (FRAP) measurement were performed using a confocal laser-scanning fluorescence microscope (FV1000-IX81, Olympus, Japan) with a 40× objective lens (LUMPLFLN40XW, Olympus).

A pipette was set up in a custom-made well composed of polydimethylsiloxane (PDMS) and a cover glass. The well was filled with 100 μL DPBS and placed on the stage of the confocal microscope. The liposome solution was added to the well and rested for 20 min to allow the lipid membrane to form on the pipette tip. After washing the excess liposomes with DPBS, the pipette tip was observed using 458 nm excitation and 476 nm emission. For exosomes, the labeled exosome solution was added into the well and observed using 633 nm excitation and 688 nm emission. No voltage was applied during the coating nor the introduction of exosomes. In the FRAP measurement, a photobleaching area of 4 μm circle was set at the tip of the nanopipette. Three images were taken before photobleaching, and then the tip was photobleached for 300 or 400 ms. A total of 70 images were acquired at 2 s intervals.

## Supplementary Material



## Data Availability

The data supporting this article are included in the Supporting Information.

## Notes

Size estimation of the pipette diameter; characterization of liposomes and milk exosomes; FRAP Analysis; fluidity of the coated lipid membranes; control experiment on recording artifacts of residual liposomes; statistic analysis of exosome translocation under different voltages; capture rates of exosomes with coated and uncoated pipettes; exosome translocation under different ionic strength; baseline stability of uncoated and coated pipettes; successful translocations and clogging events of exosomes; events on different types of lipid-membrane coatings; atatistical analysis of translocation signals of exosomes; fitting; base line and threshold settings (PDF)
