# Supplementary material for: Lipid Membrane-Coated Nanopipettes for Enhanced Resistive Pulse Sensing of Exosomes
Source: ACS Appl Mater Interfaces. 2026 Mar 23;18(13):19937–45. doi: 10.1021/acsami.6c02193 (PMC13067232; doi:10.1021/acsami.6c02193)
Supplement: Supplementary file 1 [file am6c02193_si_001.pdf]

Supporting Information for

Lipid Membrane-Coated Nanopipettes for Enhanced Resistive Pulse Sensing of Exosomes

Kaoru Hiramoto,*[a,b] Chisae Kaji,[c] and Ayumi Hirano-Iwata [b,d]

a. Frontier Research Institute for Interdisciplinary Sciences, Tohoku University, 980-8578, Aramaki-aza-Aoba, Sendai, Japan.

b. Research Institute of Electrical Communications, Tohoku University, 980-8577, Katahira, Sendai, Japan.

c. School of Engineering, Tohoku University, 980-8579, Aramaki-aza-Aoba, Japan.

d. Advanced Institute for Materials Research, Tohoku University, 980-8577, Katahira, Sendai, Japan.

*Corresponding author: kaoru.hiramoto.b4@tohoku.ac.jp (K.H.)

1. Size estimation of the pipette diameter

The diameter of nanopipette tips was estimated according to the following equation[1] :

$$R_p = \frac{1}{\kappa \pi r_i \tan \beta} + R_{access} \approx \frac{1}{\kappa \pi r_i \tan \beta} + \frac{1}{4 \kappa r_i} \qquad (S1)$$

where R_p is a nanocapillary resistance, r_i the inner capillary radius, κ is solution conductivity (1.6 S/m for DPBS), and β is the inner nanocapillary half cone angle. R_p was calculated from IV curves obtained by recording the current in 0.1 V increments from -0.6 to 0.6 V for 0.5 s each. β was measured with a bright-field microscopy image of the capillary tip shown in Table S1. The diameter for the typical n = 16 pipettes was 226±33 nm.

Table S1 The parameters used to estimate the pipette size

No.	β	1/Rp	pore size (nm)
1	8.0	125	196
2	5.1	116	276
3	5.4	88	200
4	5.6	75	166
5	5.8	106	225
6	5.2	95	224
7	7.0	138	245
8	5.7	112	241
9	5.1	84	200
10	5.5	113	251
11	6.1	115	232
12	6.3	96	187
13	5.2	112	263
14	5.0	112	275
15	5.7	117	252
16	6.9	98	178
Average			226

2. Characterization of liposomes and milk exosomes

The diameters and zeta potentials of liposomes and milk exosomes were measured with Zetasizer (Malvern Panalytical, United Kingdom) according to the manufacturer instructions (Table S2).

Table S2. Diameters and zeta potentials of milk exosomes and liposomes

	Zeta potential (mV)	Mean diameter (nm)
Milk exosome	-10±3.5	174
DOPC	2.1±0.9	76.4
30% DOTAP	24±1.5	84.2
30% DOTAP + Chol	29±0.8	115

3. FRAP analysis

The diffusion coefficient of the lipid membranes was calculated by fitting the fluorescence recovery curve using an open software.

FRAPAnalyser:https://c4science.ch/w/bioimaging_and_optics_platform_biop/teaching/frap-analysis/#frap-analyser.

Briefly, the intensity of the photobleached region was normalized using single normalization with full scale calibration by following equation.

Single normalization:

$$I_{norm}(t) = \frac{I_{frap}(t) - I_{back}(t)}{I_{frap-pre}(t)} \qquad (S2)$$

Full scale calibration:

$$I_{norm1}(t) = \frac{I_{norm}(t) - I_{norm}(t_{bleach})}{I_{norm-pre}(t) - I_{norm}(t_{bleach})} \qquad (S3)$$

where

$I_{norm}(t)$ is normalized intensity,

$I_{frap}(t)$ is measured average intensity inside the bleach spot,

$I_{back}(t)$ is measured average background intensity outside the bleach spot,

$I_{frap-pre}(t)$ is measured average intensity of the bleach spot before bleach moment after subtraction of background intensity, and

t_{bleach} is the bleach time

Then, the diffusion model for circular spot as expressed by following equation was applied[2].

$$FRAP(t) = a_0 + a_1 \cdot e^{\frac{\tau}{2(t-t_{bleach})}} \cdot \left[I_0\left(\frac{\tau}{2(t-t_{bleach})}\right) + I_1\left(\frac{\tau}{2(t-t_{bleach})}\right)\right], \qquad \tau = \frac{w^2}{D} \quad (S4)$$

Here $I_0(x)$ and $I_1(x)$ are the modified Bessel functions. Parameters are the radius of the bleached spot (*w*) and the Diffusion coefficient (*D*). Two normalizing coefficients (a_0, a_1) are introduced to account for the non-zero intensity at bleach moment and incomplete recovery.

4. Fluidity of the coated lipid membranes

The fluidity of the lipid membranes on the pipette tip was evaluated by FRAP measurement and summarized in Fig. S1 and Table S3.

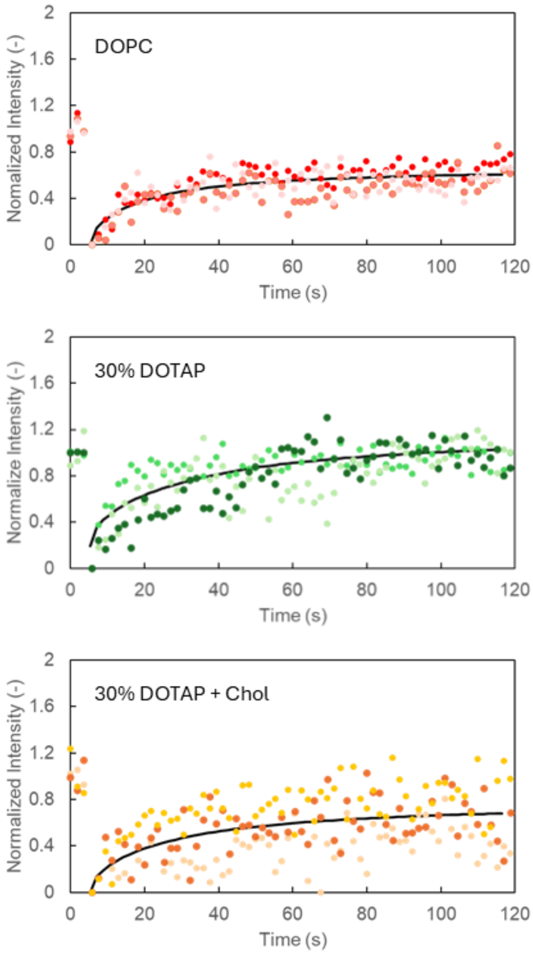

Figure S1. Fluorescence recovery curves of the tips coated with DOPC, 30% DOTAP, and 30% DOTAP + Chol membranes, respectively. FRAP measurements were taken at three locations for each lipid membrane. The solid line indicates the fitting curve.

Table S3 Diffusion coefficient of the lipid membranes coated on the pipette tips

	Diffusion coefficient ($\mu m^2/s$)
DOPC	0.52±0.02
30% DOTAP	0.29±0.15
30% DOTAP + Chol	0.34±0.04

5. Control experiment on recording artifacts of residual liposomes

To exclude possible contributions from residual coating liposomes, control experiments were performed following the lipid membrane coating procedure.

After membrane coating, the nanopipettes were thoroughly washed with DPBS to remove excess lipid vesicles. Representative current traces recorded before coating, during coating, and after washing are shown in Fig. S2. During the coating process, transient current fluctuations and blockade-like signals were observed, attributable to liposome interactions with the nanopore. However, after the washing procedure, the current trace became stable and no detectable translocation-like events were observed. Importantly, continuous current monitoring performed 60 min after washing also showed a stable baseline without spontaneous blockade events. These results indicate that residual liposomes or dissociated lipid molecules did not contribute to detectable translocation signals under the measurement conditions.

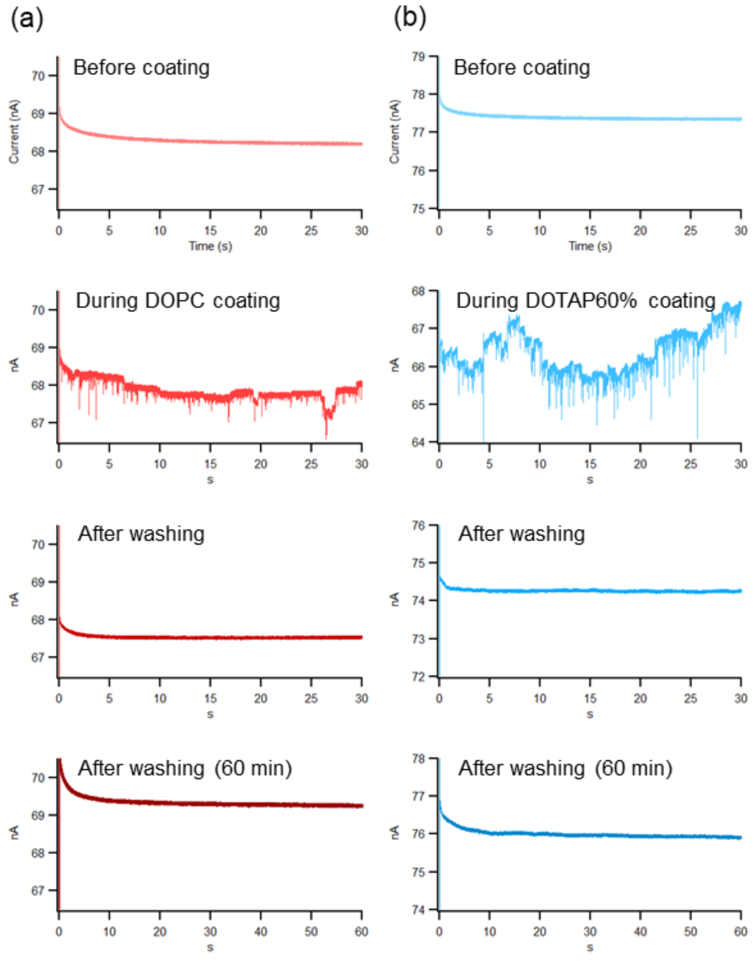

Figure S2. Representative current traces recorded before coating, during lipid membrane coating, and after thorough washing with DPBS. (a) DOPC-coated pipette, (b) 60% DOTAP coated pipette.

6. Statistical analysis of exosome translocation under different voltages

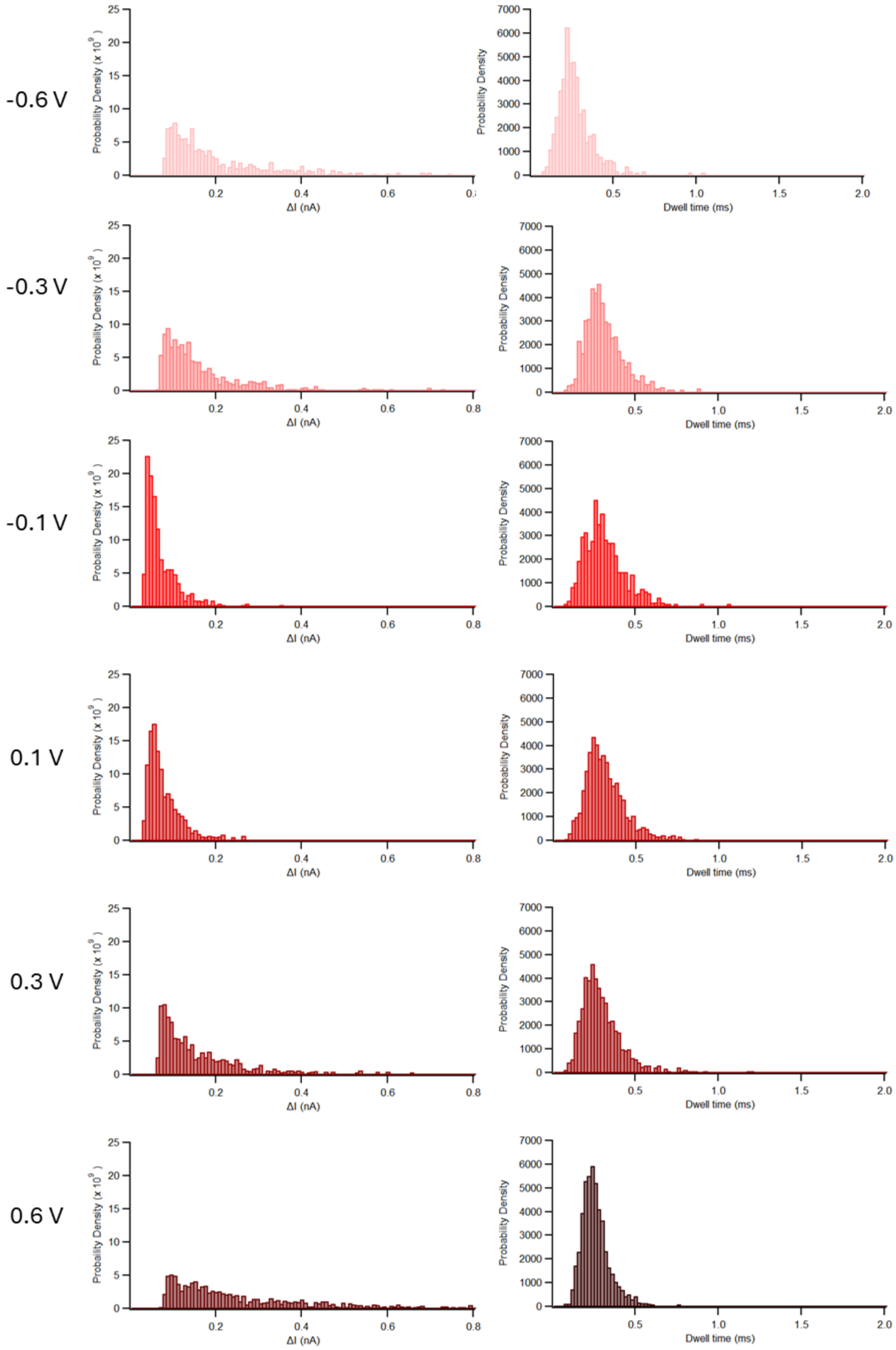

Figure S3. Histograms of ΔI/I and dwell time of exosome translocation using DOPC-membrane coated pipettes.

7. Capture rates of exosomes with coated and uncoated pipettes

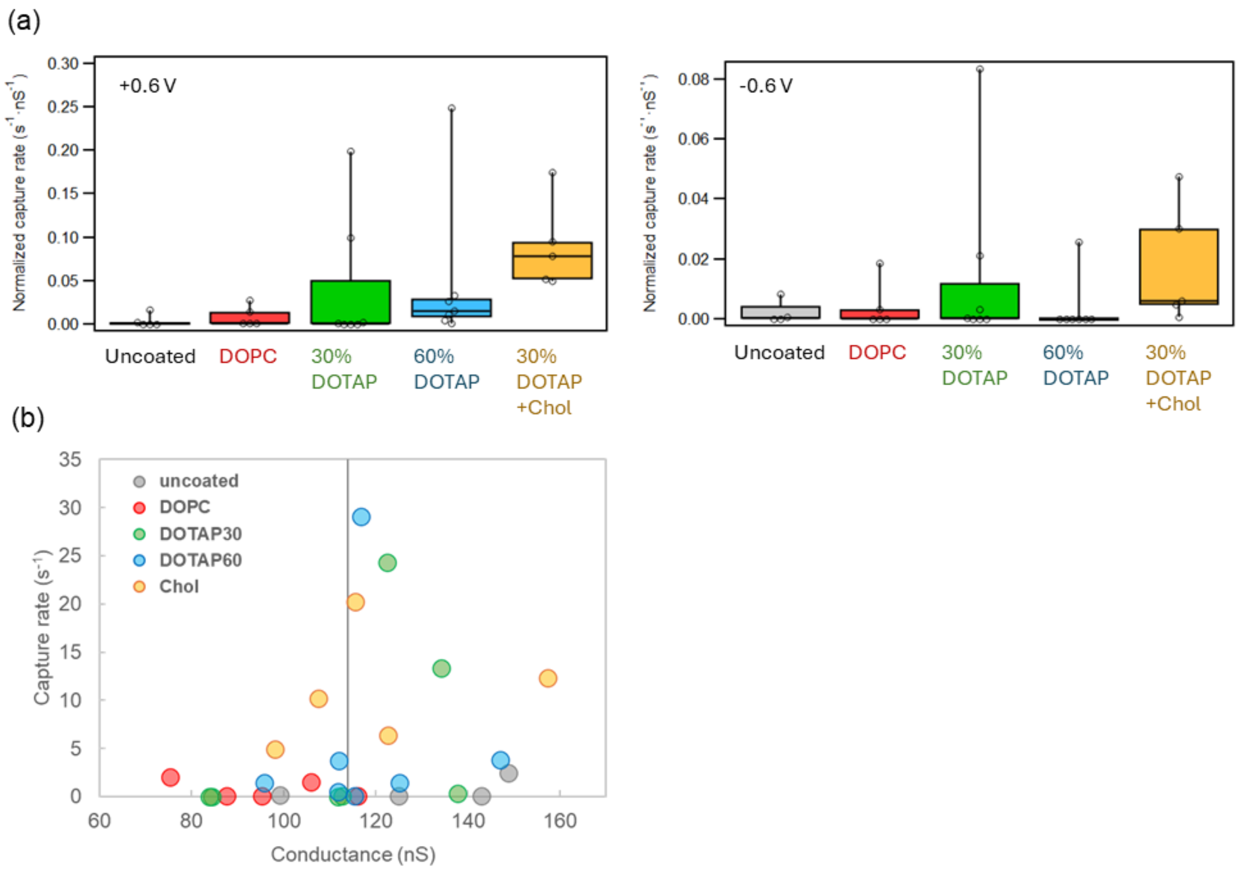

Figure S4. (a) Normalized capture rates of exosomes obtained using uncoated and lipid membrane–coated nanopipettes. The capture rate was determined by normalizing the number of signal events recorded over 60 s for each measurement. For conductance-normalized analysis, capture rates were further divided by the individual nanopipette conductance (G), which was derived from I–V measurements performed prior to each recording and used as a proxy for effective tip diameter. (b) Stratified analysis of capture rate as a function of nanopipette conductance (G). The gray line indicates the median conductance (G = 114), separating low- and high-conductance bins. DOTAP and cholesterol containing lipid membrane exhibited enhanced capture rates across both size ranges, confirming that the observed trend is not driven by pore-size variability.

8. Exosome translocation under different ionic strength

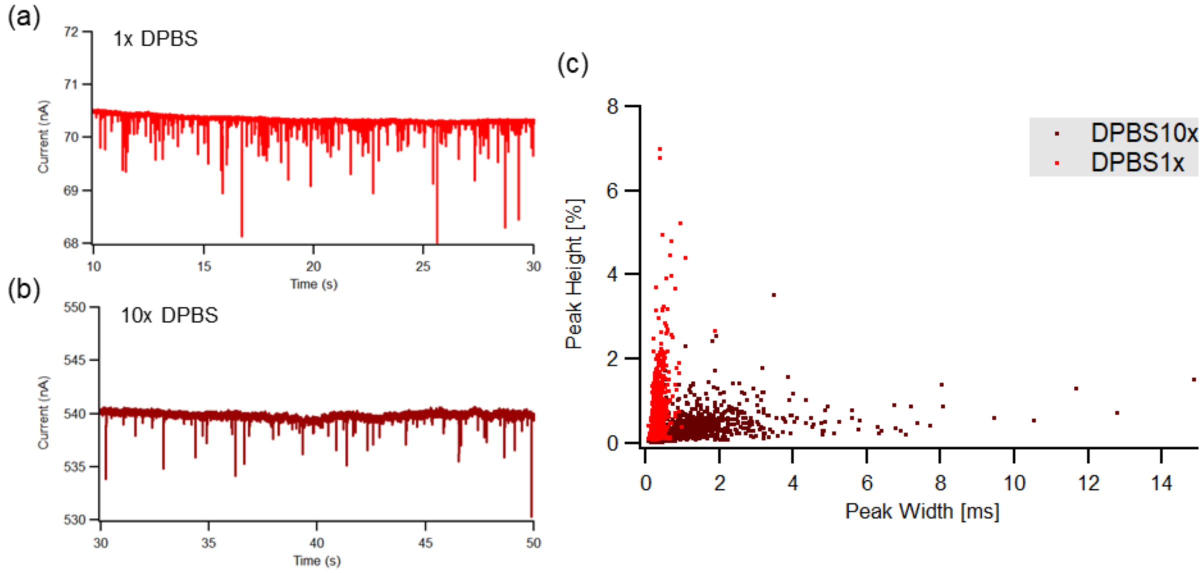

Figure S5. Exosome translocation under different ionic strength using DOPC-coated nanopipettes (low salt: 1× DPBS; high salt: 10× DPBS). (a) Current traces under 1x DPBS and (b) 10x DPBS. (c) Scatter plot of dwell time vs $\Delta I/I$ under 1x and 10x DPBS.

9. Baseline stability of uncoated and coated pipettes

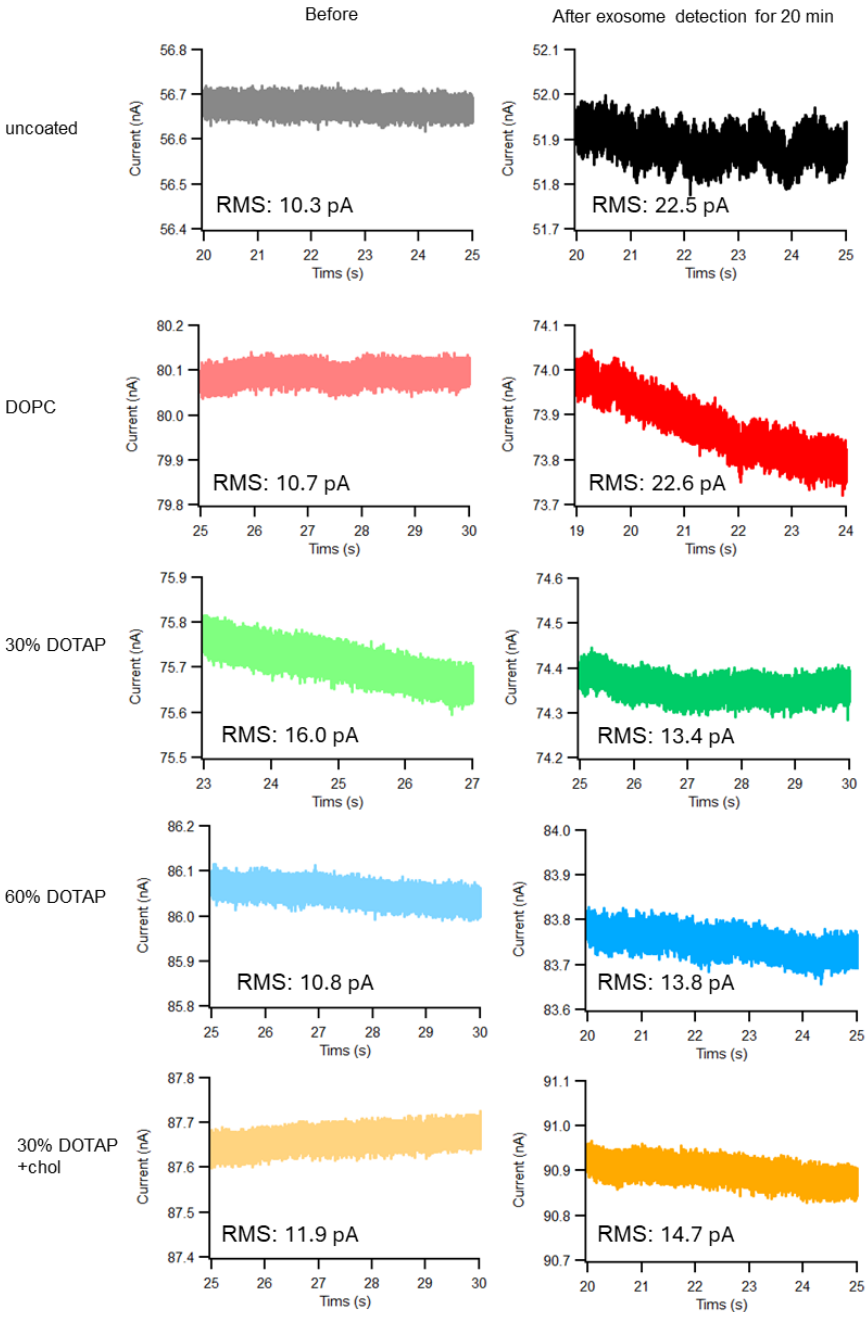

Figure S6. Current baseline stability of uncoated and lipid membrane–coated nanopipettes. Baseline currents were recorded for 60 s before exosome detection (left panels), followed by introduction of the exosome-containing solution. Recordings at +0.6 and −0.6 V were repeated for 60 s over 10–15 cycles. After approximately 20 min of measurements, the solution was replaced with pure buffer (DPBS), and the baseline current was recorded again (right panels). The baseline noise (RMS) was calculated as the standard deviation of the current trace.

10. Successful events and clogging events of exosomes

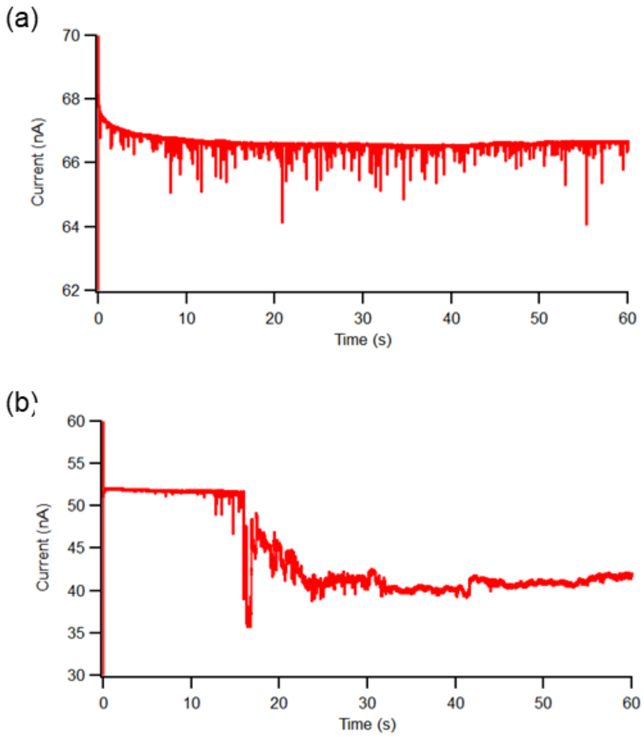

Figure S7. Representative examples of successful current recording (a) and a clogging event (b) on a DOPC-coated nanopipette. Measurements were typically performed for 60 s over 10 recording cycles. Clogging events were occasionally observed even with lipid membrane–coated nanopipettes and occurred randomly; such recordings were excluded from the statistical analysis

11. Events on different types of lipid-membrane coating

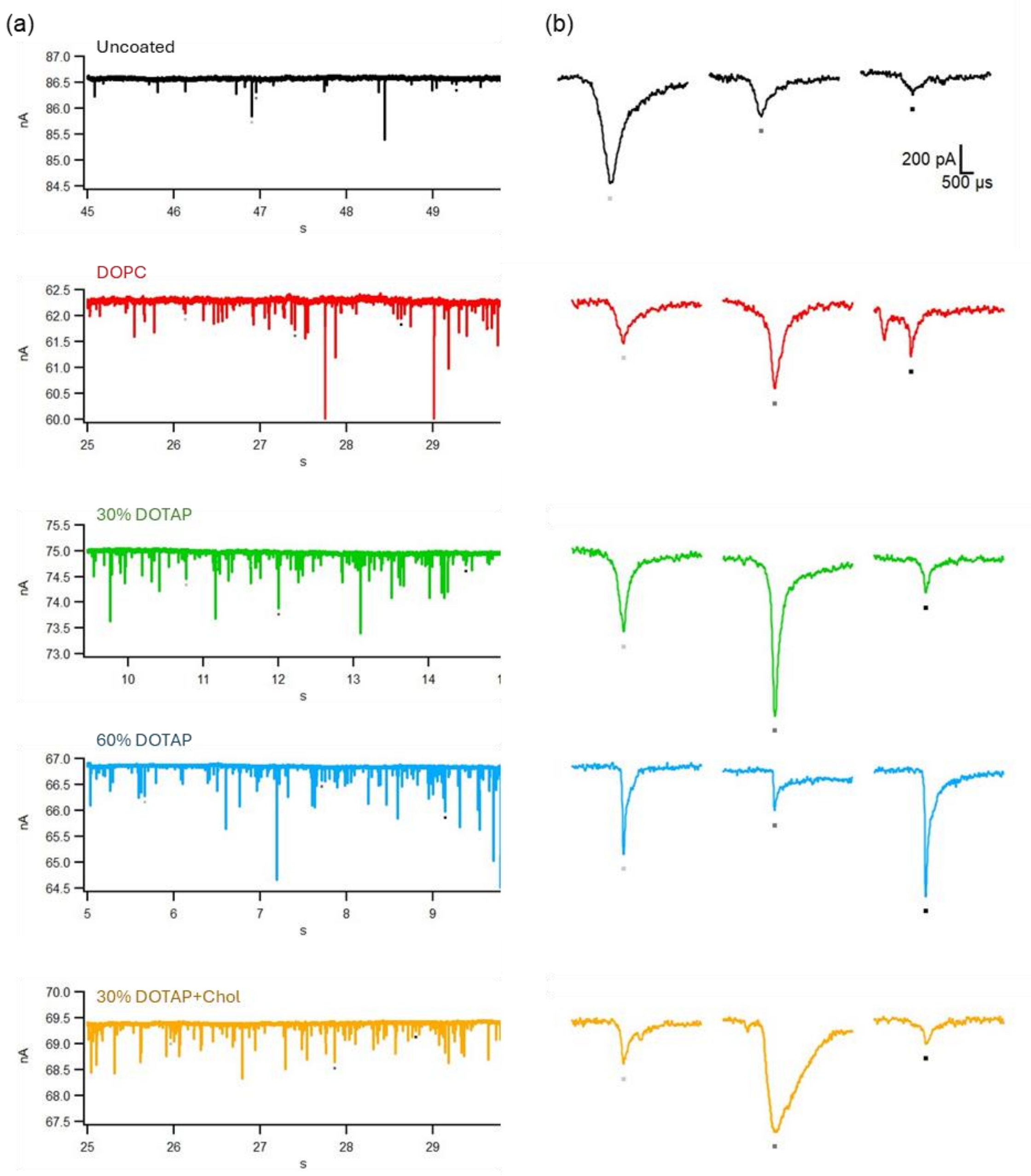

Figure S8. Typical current traces obtained from uncoated and lipid membrane-coated nanopipettes. (a) Translocation events observed in each nanopipette under an applied voltage of V_{in} = 0.6 V. (b) Representative waveforms corresponding to the events marked as dots in (a).

12. Statistical analysis of translocation signals of exosomes

Under negative voltage conditions (V_{in} = –0.6 V), translocations most likely occurred from inside the pipette to the outside, as the experiments were conducted by first applying a positive voltage and then switching to a negative voltage. Like the case under the positive potential, improvement in signal amplitude was observed except for 30% DOTAP + Chol. As there were much more time for the exosomes to interact with inner surface wall during the passage from in to out, the dwell time increased for DOTAP-containing lipid compositions, consistent with enhanced electrostatic interactions between the positively charged membrane and the negatively charged exosomes. The dwell-time distributions also broadened significantly, particularly for 60% DOTAP and 30% DOTAP + cholesterol, and were well fitted by a Gaussian distribution. Such fluctuations likely arise from stochastic electrostatic interactions with the DOTAP-coated inner pipette wall and from the local packing effect of cholesterol.

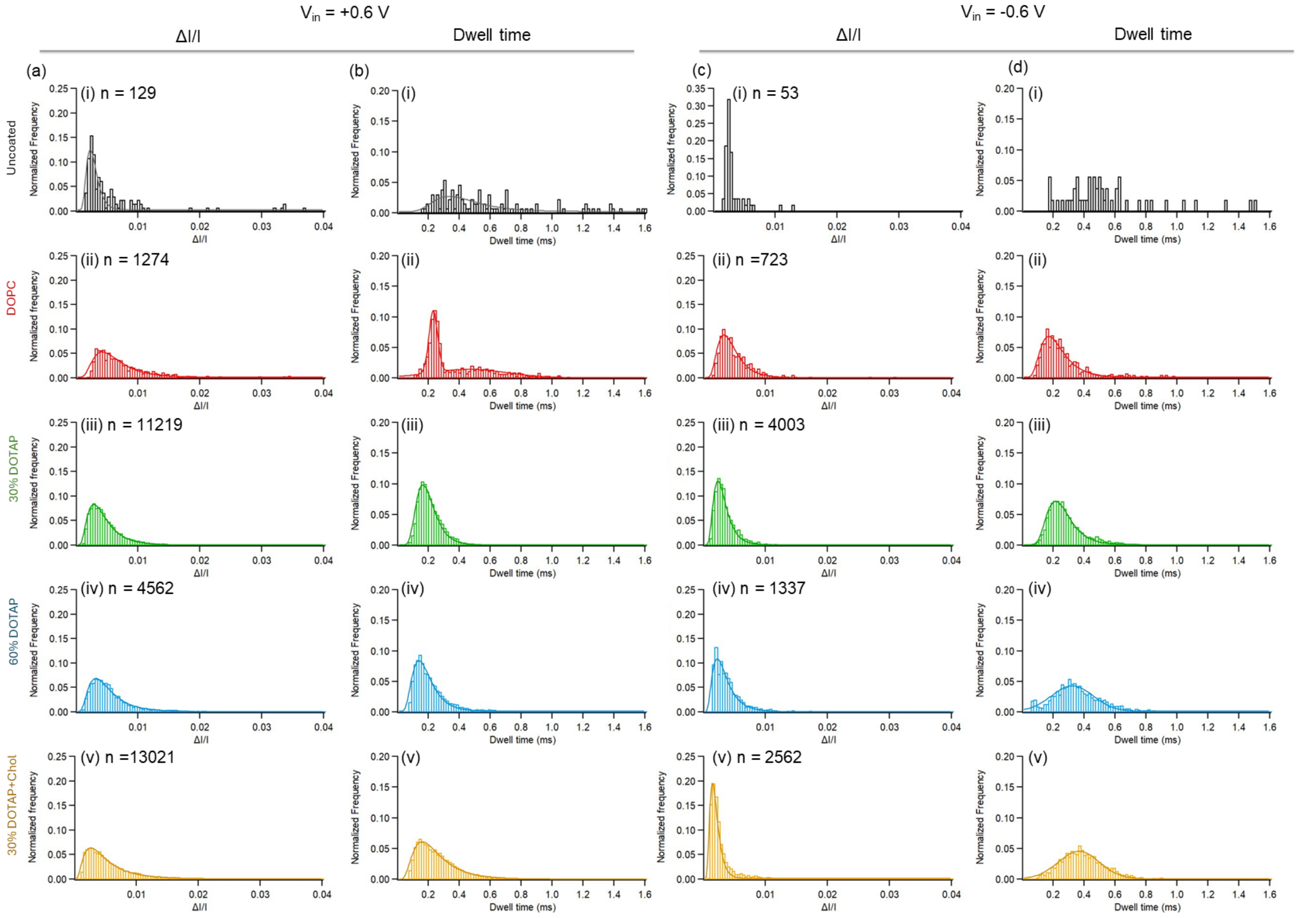

Figure S9. Statistical analysis of exosome translocations with uncoated and lipid membrane coated nanopipettes. (a-d) Histograms showing the trend of normalized peak height (ΔI/I) (bin-width: 0.004) and dwell time (bin-width: 16 μs) of translocation signals under V = +0.6 V (a, b) and -0.6 V (c, d).

13. Fitting

For Figs 6a and c, the peak amplitude was normalized as below:

$$\frac{\Delta I}{I} = \frac{I_0 - I_{peak}}{I_0} \qquad (S5)$$

where I_0 is a baseline current and I_{peak} is a maximum current extracted from a translocation event.

Most histograms of ΔI/I and dwell time exhibited pronounced right-hand tails, characteristic of non-Gaussian distributions. This behavior indicates that translocation is governed by multiple stochastic interactions within the nanopore, such as transient adsorption and desorption of exosomes at the lipid membrane wall, as well as modulation of electroosmotic flow (EOF) induced by the lipid coatings. Accordingly, the most probable values of ΔI/I and the dwell times (defined as the full width at half maximum, FWHM) were determined by fitting the histograms with a log-normal distribution described by the following equation:

$$y = y_0 + A\exp\left\{-\left(\frac{ln(x/x_0)}{w}\right)^2\right\} \qquad (S6)$$

where $y_0$is the baseline, A is the peak amplitude, x_0 represents the most probable value, and $w$is the width parameter.

Exceptions were observed for the dwell-time distributions obtained with 60% DOTAP and 30% DOTAP + cholesterol at an applied voltage of 0.6 V (Figs. 6d(v) and (vi)), which were better described by a Gaussian distribution given by:

$$y = y_0 + A\exp\left\{-\left(\frac{x - x_0}{w}\right)^2\right\} \qquad (S7)$$

In contrast, the histogram of ΔI/I obtained with the DOPC-only membrane under −0.6 V was more complex and could not be adequately described by a single distribution. Instead, it was best fitted by a two-Gaussian model:

$$y = y_0 + A_1\exp\left\{-\frac{(x - x_1)^2}{2{w_1}^2}\right\} + A_2\exp\left\{-\frac{(x - x_2)^2}{2{w_2}^2}\right\} \qquad (S8)$$

This bimodal behavior indicates that the DOPC-only coating is likely less uniform and less stable, resulting in multiple translocation pathways with distinct blockade characteristics.

14. Base line and threshold settings

To construct the baseline, the recorded current traces were first processed using a 1 kHz low-pass filter designed with a Hamming window (101 coefficients), with the end of the passband at 0.5 kHz and the start of the stopband at 1 kHz. This 1 kHz filter was used exclusively for baseline estimation. For event detection, a separate 10 kHz low-pass filter was applied.

After filtering, the baseline was defined using a moving median. A window size of 500 data points (5 ms at a 100 kHz sampling rate) was used for standard measurements. In experiments comparing low-salt and high-salt conditions—where slow baseline drift was observed in the high-salt case—a larger 5000-point window (50 ms) was used to ensure accurate baseline tracking without distorting transient events.

Following baseline subtraction, the current trace was divided into 5 s segments for event detection. Within each segment, the noise level was defined as the maximum absolute current fluctuation in the direction opposite to translocation events. A translocation event was identified when the corrected current exceeded a threshold of 1.3 × the noise level. This multiplier was selected to balance sensitivity and suppression of false positives (Fig. S10).

The peak amplitude was normalized to the instantaneous baseline current to obtain the normalized peak height. The full width at half maximum (FWHM) was defined as the peak width and used as the translocation dwell time.

Events with a normalized peak height >10% or peak width >1 ms were classified as outliers and excluded from statistical analysis to avoid inclusion of adsorption-related artifacts. No automated deconvolution algorithms were applied and no manual curation was performed.

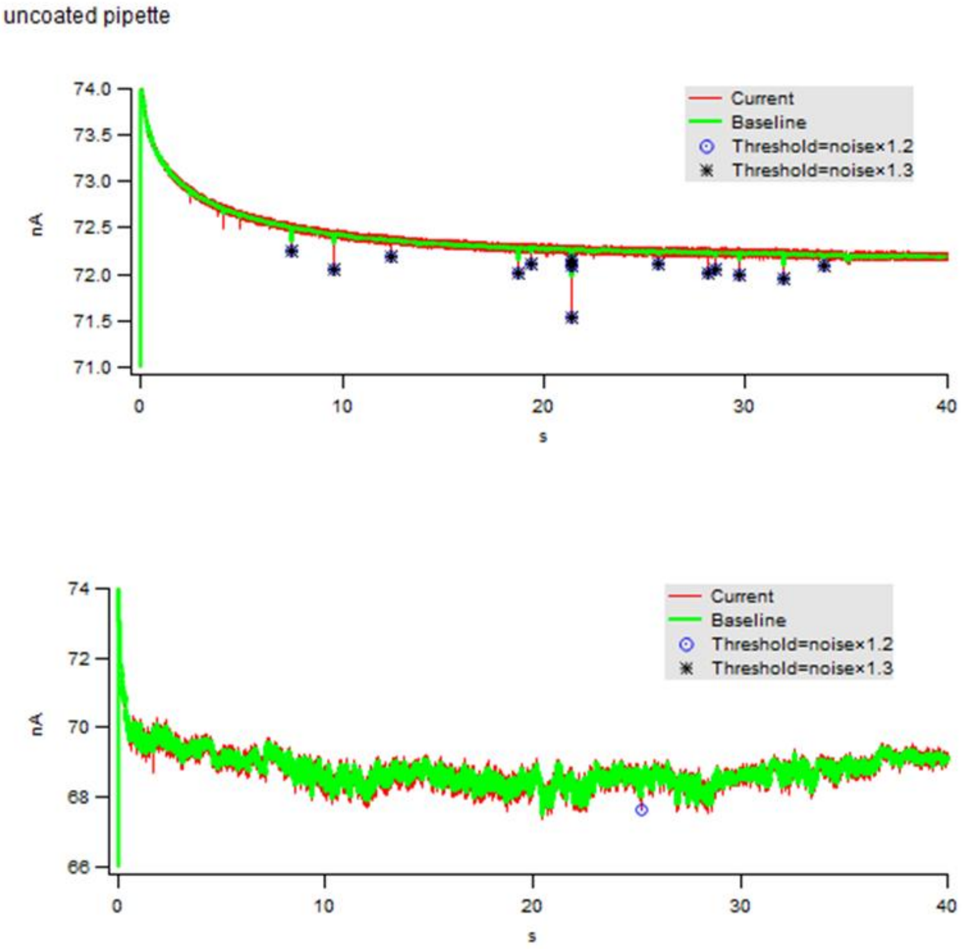

Figure S10. Comparison of event detection thresholds set at 1.3× and 1.2× the baseline noise level. Under stable and smooth baseline conditions, both thresholds detected essentially the same number of translocation events. However, when baseline fluctuations occurred due to exosome adsorption, the 1.2× threshold identified additional noise-like spikes as events. Therefore, a threshold of 1.3× the noise level was selected to reduce false-positive detections while maintaining sufficient sensitivity.

References
(1) Perry, D.; Momotenko, D.; Lazenby, R. A.; Kang, M.; Unwin, P. R. Characterization of Nanopipettes. Anal. Chem. 2016, 88 (10), 5523-5530.
(2) Soumpasis, D. M. Theoretical Analysis of Fluorescence Photobleaching Recovery Experiments. Biophys. J. 1983, 41 (1), 95-97. Pincet, F.; Adrien, V.; Yang, R.; Delacotte, J.; Rothman, J. E.; Urbach, W.; Tareste, D. Frap to Characterize Molecular Diffusion and Interaction in Various Membrane Environments. PLoS One 2016, 11 (7), e0158457.
